# Clinical characteristics and associated factors of pediatric acute disseminated encephalomyelitis patients with MOG antibodies: a retrospective study in Hangzhou, China

**DOI:** 10.1186/s12883-022-02963-0

**Published:** 2022-11-09

**Authors:** Jue Shen, Donghui Lin, Tiejia Jiang, Feng Gao, Kewen Jiang

**Affiliations:** 1 Department of Neurology, Children’s Hospital, School of Medicine, Zhejiang University, 3333 Binsheng Road, Binjiang District, Hangzhou City, 310052 P.R. China; 2 Department of Child Psychology, Children’s Hospital, School of Medicine, Zhejiang University, 3333 Binsheng Road, Binjiang District, Hangzhou City, 310052 P.R. China; 3 Department of Biobank, Children’s Hospital, School of Medicine, Zhejiang University, 3333 Binsheng Road, Binjiang District, Hangzhou City, 310052 P.R. China; 4 Department of Neurobiology, Key Laboratory of Medical Neurobiology of Ministry of Health of China, Zhejiang Province Key Laboratory of Neurobiology, School of Medicine, Zhejiang University, 3333 Binsheng Road, Binjiang District, Hangzhou City, 310052 P.R. China

**Keywords:** Acute disseminated encephalomyelitis, Myelin oligodendrocyte glycoprotein antibodies, Children, Encephalopathy, Associated factors

## Abstract

**Background:**

To explore the clinical characteristics and related factors of children with acute disseminated encephalomyelitis (ADEM) with positive anti-myelin oligodendrocyte glycoprotein (MOG) antibody.

**Methods:**

A retrospective study was conducted and enrolled pediatric ADEM patients who underwent serum MOG antibody detection from May 2017 to August 2020. The patients were divided into two groups: MOG- immunoglobulin G (IgG) positive (*n* = 35) and MOG-IgG negative (*n* = 50). We analyzed the clinical characteristics of MOG-IgG-positive ADEM pediatric patients and conducted a comparative analysis between the two groups.

**Results:**

Thirty-five patients (21 males and 14 females) in the MOG-IgG-positive group with encephalopathy, multifocal neurological symptoms, and typical magnetic resonance imaging (MRI) abnormalities were enrolled. They usually had a favorable outcome, while some suffered from relapse. Compared to the MOG-IgG-negative group, MOG-IgG-positive ADEM patients had a longer disease duration (median: 10 vs. 6 days), more meningeal involvement (31.4% vs. 8%) and frontal lobe involvement (82.8% vs. 68%), higher relapse rates (14.3% vs. 2%), lower serum tumor necrosis factor (1–12.4 pg/ml, median 1.7 vs. 1–34 pg/ml, median 2.2) and interferon-gamma (1–9.4 pg/ml, median 1.3 vs. 1–64 pg/ml, median 3) (*P* < 0.05, respectively). Multivariate logistic regression analysis showed that the longer disease duration, meningeal involvement and frontal lobe involvement were the correlated factors of patients with ADEM with MOG antibody (*P* < 0.05).

**Conclusions:**

Our findings provide clinical evidence that MOG-IgG positivity is associated with longer disease duration, meningeal involvement, and frontal lobe involvement.

## Background

Myelin oligodendrocyte glycoprotein (MOG) antibody, expressed in the outermost layer of the myelin sheaths in the mammalian central nervous system (CNS), mediates a variety of demyelinating diseases [1]. MOG-immunoglobulin G (IgG)-associated disorders (MOGADs) have a variety of clinical phenotypes and have been the focus of neurology research in recent years. The first episode of MOGADs in children involves acute disseminated encephalomyelitis (ADEM) and optic neuritis (ON) [2], and ADEM is particularly common in young children [3]. ADEM is an immune-mediated demyelinating CNS disease, manifested as encephalopathy, polyfocal neurologic symptoms, and multifocal demyelinating lesions in the brain and spinal cord [4]. Most children with ADEM have a good prognosis, and a few may have recurrence and residual sequelae. So far, the pathological mechanism and risk factors for anti-MOG antibody-positive ADEM are still not well established. This study retrospectively compared the clinical characteristics and prognosis of ADEM children with and without MOG antibody to improve the diagnosis and treatment of MOG-IgG-positive ADEM and to identify the factors associated with this disease.

## Materials and methods

**Fig. 1:**
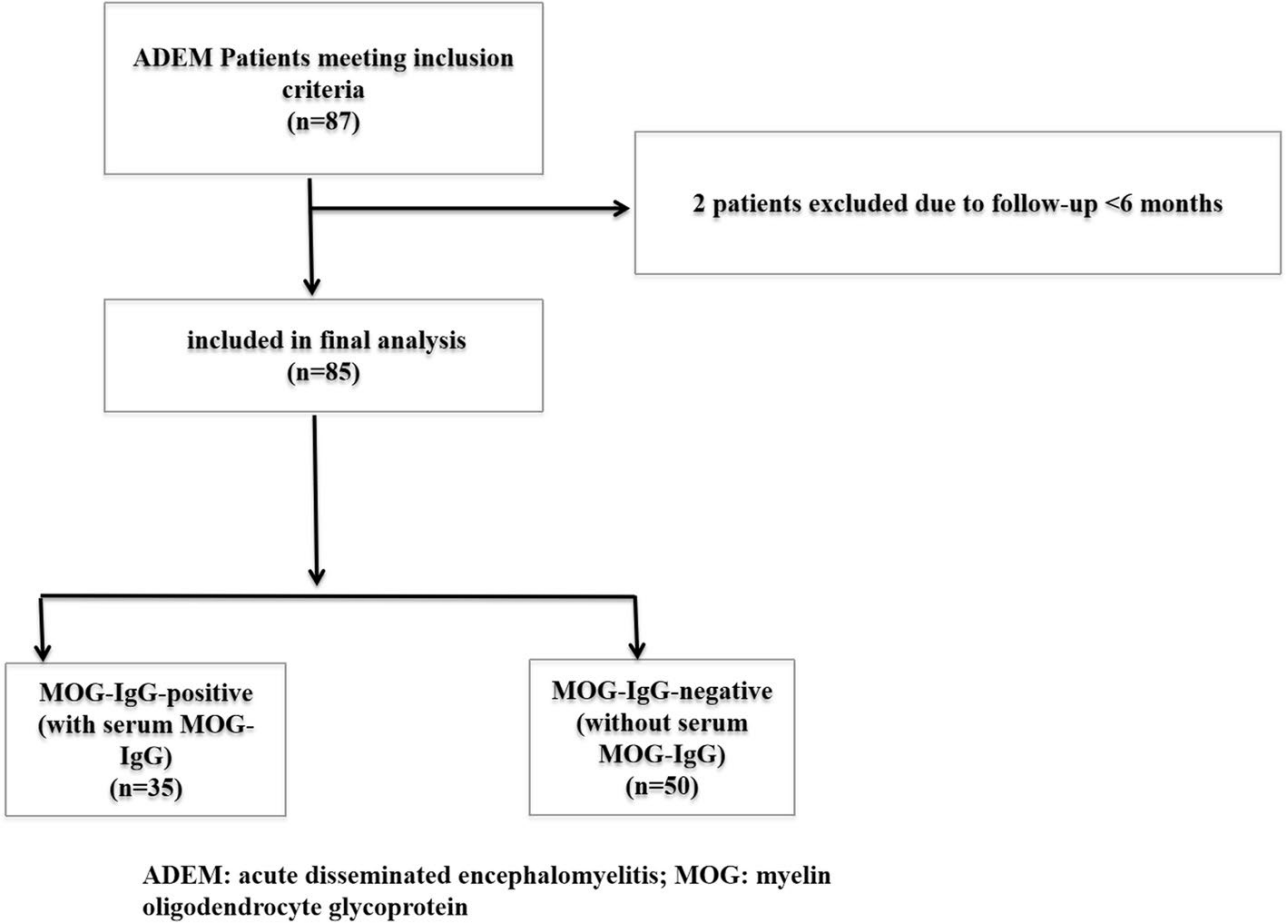
The flowchart of the participants’ recruitment process

A retrospective study was conducted in the Department of Neurology, Children’s Hospital of Zhejiang University School of Medicine, China, and included pediatric ADEM patients who underwent serum MOG antibody detection from May 2017 to August 2020. Their clinical data were collected. The study was approved by the Ethics Committee of the Children’s Hospital of Zhejiang University School of Medicine, China (2019-IRB-115). All pediatric patients provided informed consent. The MOG antibody in the serum was determined using CBAs (cell-based assays) on the first day of hospitalization, and the levels of ≥1:10 were classified as positive. According to the serum MOG antibody level, the patients were divided into two groups: MOG-IgG-positive and MOG-IgG-negative. The diagnostic criteria of ADEM were in line with the 2013 International Pediatric Multiple Sclerosis Study Group criteria for pediatric ADEM, including all of the following: (1) the first multifocal CNS event (probably caused by inflammatory demyelination); (2) symptoms of encephalopathy (disorder of consciousness or behavioral changes) that could not be explained by fever; (3) no new clinical or lesions revealed by MRI 3 months after onset; (4) abnormal head MRI in the acute stage (within 3 months); and (5) typical findings in head MRI. The exclusion criteria were (1) less than 3 months of follow-up and (2) other intracranial infectious diseases or systemic autoimmune diseases [5]. After excluding two patients who were followed up for less than three months, a total of 85 ADEM patients who underwent serum MOG-IgG testing were finally included in our cohort (Fig. 1). Thirty-five patients were included in the MOG-IgG-positive group, and the remaining 50 MOG-IgG-negative patients were included in the MOG-IgG-negative group.

All demographic, clinical, laboratory, and MRI data were collected from all patients. The initial cerebrospinal fluid (CSF) evaluations and MRI scans were performed within five days of admission. Pathogen detections (including viral polymerase chain reaction studies and bacterial culture), autoimmune encephalitis antibodies, anti-aquaporin 4 (AQP4) antibodies, anti-myelin basic protein antibodies, and cytokines were tested in the serum and CSF samples in all patients. In addition, electroencephalogram (EEG), complete blood counts, C-reactive protein, serum chemistry, complements, and erythrocyte sedimentation rates were also collected at admission. All patients were followed up at the outpatient clinic for at least one year. Follow-up brain and spinal cord MRI was performed every 3–6 months.

The SPSS 22.0 statistical software package was used in this study. The normal distribution of data was tested by the Kolmogorov-Smirnov test. Continuous variables with a normal distribution were compared using a t-test, while those that exhibited a nonnormal distribution were compared with the Mann-Whitney U-test. Categorical variables were compared with the chi-square test. Factors associated with MOG-IgG-positive ADEM were identified through a binary logistic regression model. The factors with statistical significance in univariate analysis and other potential predictor variables were included in multivariate models for analysis. The test standard was *P* = 0.05, and *P* < 0.05 was considered statistically significant.

## Results

### Clinical characteristics of ADEM children with positive anti-MOG antibody

We identified 35 patients, 21 males and 14 females (male-to-female ratio of 1.5:1), with a median onset age of 69 months (range: 20–138 months), with positive MOG antibody i.e., MOG-IgG-positive group. The disease duration ranged from 1 to 30 days (median: 10 days), with a hospital stay length of 6–35 days (median: 17 days). The time from disease onset to MOG-antibody sampling ranged from 1 to 22 days (median: 6 days). In our cohort, there was a seasonal increase in spring or winter incidence rate (13 cases in winter, 10 cases in spring, 5 cases in summer, and 7 cases in fall). Among the patients, 28.6% (10/35) had an acute upper respiratory history, while the other 71.4% of patients had no preceding or concomitant diagnosis of respiratory infection. None of our patients had a preceding vaccination history. Encephalopathy was seen in all patients, including altered consciousness in 21 patients (60%) and behavioral changes in the other 14 children (40%). Other symptoms included fever (23/35, 65.7%), headache (15/35, 42.9%), vomiting (13/35, 37.1%), limb weakness (13/35, 37.1%), meningeal irritation (11/35, 37.1%), seizures (8/35, 22.3%), optic neuritis (6/35, 17.1%) and ataxia (3/35, 8.6%) (Table 1).

**Table 1:** Clinical features of MOG-IgG-positive ADEM patients compared to MOG-IgG-negative ADEM patients

Clinical features	MOG-IgG-positive *n* = 35	MOG-IgG-negative *n* = 50	Univariate analyses *p* value
Basic Information			
Gender, male, n (%)	21 (60)	28 (56)	0.713
Onset age, months, median (range)	69 (20–138)	76 (13–178)	0.317
Disease duration, days, median (range)	10 (1–30)*	6 (0.5–30)	0.048
Hospital stay, days, median (range)	17 (6–35)	16 (7–40)	0.567
With respiratory symptoms, n (%)	10 (28.6)	17 (34)	0.771
Onset at winter or spring, n (%)	23 (65.7)	24 (48)	0.358
Clinical characteristics			
Encephalopathy, n (%)			
Altered consciousness, n (%)	21 (60)	38 (76)	0.152
Behavioral changes, n (%)	14 (40)	12 (24)	0.152
Fever, n (%)	23 (65.7)	37 (74)	0.472
Headache, n (%)	15 (42.9)	26 (52)	0.509
Vomiting, n (%)	13 (37.1)	22 (44)	0.655
Seizures, n (%)	8 (22.3)	13 (26)	0.801
Optic neuritis, n (%)	6 (17.1)	4 (8)	0.305
Meningeal involvement, n (%)	11 (31.4)*	4 (8)	0.008
Ataxia, n (%)	3 (8.6)	6 (12)	0.731
Limb weakness, n (%)	13 (37.1)	17 (34)	0.820
CSF analysis			
Pleocytosis, n (%)	29 (82.9)	32 (64)	0.858
Protein elevation, n (%)	13 (37.1)	15 (30)	0.550
Positive OB, n (%)	5 (14.3)	3 (6)	0.265
Therapeutic characteristics			
Neurological sequel, n(%)	5 (14.3)	11 (22)	0.413
Relapse, n (%)	5 (14.3)*	1 (2)	0.030
IVIG therapy, n (%)	21 (60)	35 (70)	0.339
Methylprednisolone therapy	35 (100)	50 (100)	1.000

*ADEM* acute disseminated encephalomyelitis, *MOG* myelin oligodendrocyte glycoprotein, *MOG-IgG-positive* ADEM patients with positive anti-MOG antibodies in serum, *MOG-IgG-negative* ADEM patients without positive anti-MOG antibodies in serum, *IVIG* intravenous immunoglobulin; univariate analyses were confirmed by the t-test, chi-square or Mann-Whitney U-test.

*indicates MOG-IgG-positive group vs. MOG-IgG-negative group, *p* < 0.05.

The initial cranial MRI revealed typical multifocal high lesions on fluid attenuated inversion recovery and T2-weighted images in all 35 patients in the MOG-IgG-positive group. The most commonly involved brain site was the frontal lobe (29/35, 82.9%), followed by the parietal lobe (19/35, 54.3%), temporal lobe (18/35, 51.4%) and occipital lobe (13/35, 37.1%). In addition the basal ganglia (17/35, 48.6%), thalamus (16/35, 45.7%), brainstem (13/35, 37.1%), cerebellum (9/35, 25.7%) and corpus callosum (5/35, 14.3%) were also involved. Lesions in the thalamus, basal ganglia, brainstem and cerebellum were bilaterally involved in general, and corpus callosum lesions were mainly involved in the compression part (4/5, 80%). Initial spinal cord MRI showed lesions of high signal intensity on T2-weighted images in 42.9% (15/35) of the cases, in which the cervical, thoracic, lumbar and sacral cord were invaded in 42.9% (15/35), 37.1% (13/35), 17.1% (6/35) and 5.7% (2/35) of patients, respectively (Table 2).

**Table 2:** The MRI features of MOG-IgG-positive ADEM patients compared to MOG-IgG-negative ADEM patients

MRI lesions, n (%)	MOG-IgG-positive *n* = 35	MOG-IgG-negative *n* = 50	*p* value
Brain lesions			
Frontal lobe	29 (82.8)*	34 (68)	0.019
Parietal lobe	19 (54.3)	27 (54)	0.140
Temporal lobe	18 (51.4)	23 (46)	1.000
Occipital lobe	13 (37.1)	14 (28)	0.664
Basal ganglia	17 (48.6)	22 (44)	0.825
Thalamus	16 (45.7)	14 (28)	0.380
Brainstem	13 (37.1)	14 (28)	0.478
Cerebellum	9 (25.7)	12 (24)	0.527
Corpus callosum	4 (14.3)	5 (10)	0.734
Optic nerve	6 (17.1)	4 (8)	0.305
Spinal cord lesions	15 (42.9)	29 (58)	0.662
LETM	6 (17.1)	9 (18)	0.580
Infratentorial lesions	16 (45.7)	19 (38)	0.380
Bilateral lesions	35 (100)	50 (100)	1.000
Residual cranial lesions	23 (65.7)	29 (58)	0.506
Residual spinal cord lesions	4 (11.4)	5 (10)	1.000

*ADEM* acute disseminated encephalomyelitis, *MOG* myelin oligodendrocyte glycoprotein, *MOG-IgG-positive* ADEM patients with positive anti-MOG antibodies in serum, *MOG-IgG-negative* ADEM patients without positive anti-MOG antibodies in serum, *MRI* magnetic resonance imaging, *LETM* longitudinal extensive transverse myelitis; univariate analyses were confirmed by the t-test, chi-square or Mann-Whitney U-test.

*indicates MOG-IgG-positive group vs. MOG-IgG-negative group, *p* < 0.05.

CSF analysis showed pleocytosis (≥5/μl, range 12–352/μl, monocyte predominance) in 29 (82.9%) cases and protein elevation in 13 (37.1%) cases (> 400 mg/L, range 388–972 mg/L) (Table 2). CSF glucose (normal 2.5–4.4 mmol/L, glucose ratio 0.4–0.5) and chloride (normal range, 110–122 mmol/L for infants; children 117–127 mmol/L) were normal in all patients. Intrathecal oligoclonal IgG was present in the CSF in five patients. There were 8 patients (22.9%) with MOG-IgG positivity in the CSF. The results of the autoimmune encephalitis antibodies, AQP 4 antibodies, and anti-myelin basic protein antibodies in the CSF and serum were negative in all children. Interictal EEGs demonstrated that 23 patients (65.7%) had abnormal background activity, and none showed epileptiform discharges. Six patients showed abnormal visual evoked potentials. All children had normal brainstem auditory evoked potentials. The median serum MOG-IgG titre was 1:20 (range 1:10–1:320). We also studied the correlation between serum MOG-IgG titre and ADEM recurrence. Spearman’s test was used, and the significance level was 5%. The results showed that no correlation was observed between the two variables (*r* = 0.051), indicating that the patient’s MOG status could not predict polyphasic cases.

All children received high-dose, intermittent intravenous methylprednisolone therapy (20–30 mg/kg/day, 5 days for 1 course, 1–2 courses in total) followed by tapered oral corticosteroids over 6–8 weeks, whereas 21 patients received intravenous immunoglobulin therapy (2 g/kg divided over 2 days, according to the recommendation of the references [6]: IVIG is usually administered over a course of 1–5 days with total dosage of 1–2 g/kg, not exceeding 1 g/kg/day), and 6 cases (17.1%) were treated with immunosuppressants (3 azathioprine, 2 mycophenolate mofetil and 1 rituximab). All clinical symptoms and signs were significantly improved within 1–3 weeks of the treatment. With a median follow-up of 1.6 (range, 1.2–2.3) years, 30 cases (85.7%) were diagnosed with monophasic ADEM, while the remaining 5 cases (14.3%) recurred during follow-up. Among the five recurrent cases, the final diagnoses were multiphasic disseminated encephalomyelitis (MDEM, *n* = 3), neuromyelitis optica spectrum disorders (NMOSDs, *n* = 1), and ADEM-ON (*n* = 1). All recurrent episodes occurred between 4 to 10 months after the initial event. Five patients (14.3%) suffered from neurological sequelae, including motor deficits (*n* = 3) and secondary epilepsy (*n* = 2). Five recurrent patients showed new MRI lesions during the follow-up period between 4 to 10 months, and the lesions were significantly improved after subsequent retreatment. Nevertheless, MRI studies at one-year follow-up showed that the lesions in the brain and spinal cord were improved to varying degrees. Brain MRI scans showed complete T2 lesion resolution in 12 patients (34.3%) and partial reduction in the size of the T2 lesion in 23 patients (65.7%), while spinal cord MRI scans showed complete T2 lesion resolution in 31 patients (88.6%). Of the 23 patients with abnormal EEG, the recheck EEG showed that only five cases were mildly abnormal, and the remaining 18 cases recovered to a normal level at the one-year follow-up. At the most recent follow-up, 18 patients (51.4%) were still positive for MOG-IgG in the serum (median 1:20; range 1:10–1:100). We compared the clinical characteristics and prognosis between the persistently and transiently positive MOG-IgG patients, and no significant differences in fever (10/18 vs. 13/17, *p* = 0.193), headache (8/18 vs. 7/17, *p* = 0.845), seizure (5/18 vs. 3/17, *p* = 0.476), meningeal involvement (4/18 vs. 7/17, *p* = 0.227), neurological sequelae (2/18 vs. 3/17, *p* = 0.679), or relapse (4/18 vs. 1/17, *p* = 0.167) were identified.

### Comparison of clinical, MRI, and laboratory features between the MOG-IgG positive and negative groups

**Fig. 2:**
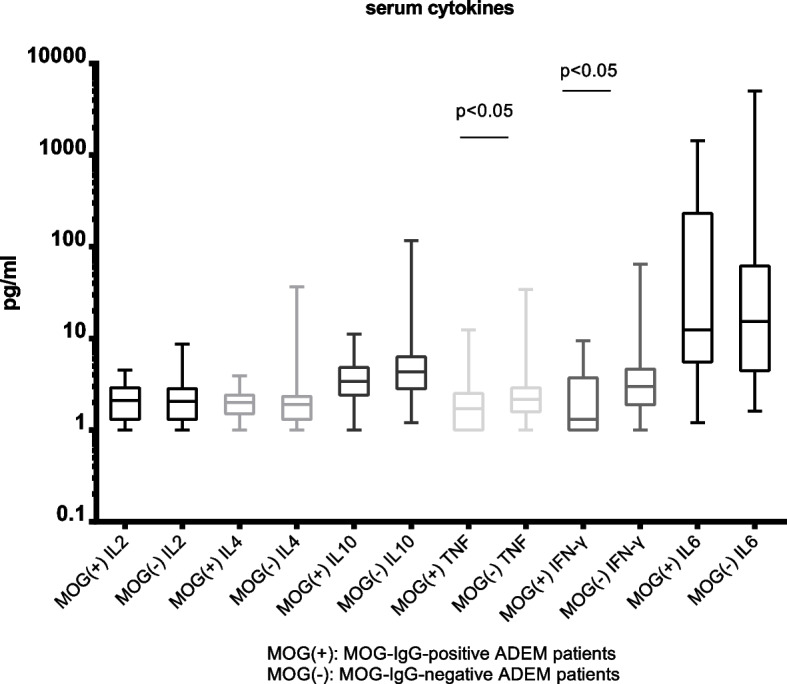
The serum cytokines characteristics of MOG-IgG-positive ADEM patients compared to MOG-IgG-negative ADEM patients

To evaluate what characterizes ADEM children with the positive MOG antibody, a statistical comparison of clinical data, including the general conditions, clinical manifestations, imaging manifestations, laboratory findings, and prognosis after treatment, was performed between the MOG-IgG positive and negative groups (Tables 1, 2). The results showed that the MOG-IgG-positive group had a significantly longer disease duration (median: 10 vs. 6 days, *P* < 0.05), more meningeal involvement (31.4% vs. 8%, *P* < 0.05), and a higher recurrence rate (14.3% vs. 2%, *P* < 0.05) than that of the MOG-IgG-negative group (Table 1). In comparison of the initial and follow-up MRI studies between the two groups revealed that MOG-IgG positive patients had a higher chance of frontal lobe involvement (82.8% vs. 68%, *P* < 0.05), in contrast to children with absent MOG antibody. Another finding was that children with or without MOG antibody did not differ in bilateral lesions, spinal cord involvement, infratentorial lesion involvement, or residual MRI findings. Furthermore, the serum levels of tumor necrosis factor (TNF) and interferon γ (IFN-γ) in the MOG-IgG-positive group were significantly lower than those in the MOG-IgG-negative group (TNF: 1–12.4 pg/ml, median 1.7 vs. 1–34 pg/ml, median 2.2, *P* < 0.05; IFN-γ: 1–9.4 pg/ml, median 1.3 vs. 1–64 pg/ml, median 3, *P* < 0.05) (Fig. 2), while no significant differences in serum interleukin (IL)-2, 4, 6, 10, CSF cytokines, or serum complements were noted between the two groups. In addition, no significant differences in age, gender, other clinical characteristics, or other laboratory findings were found between the two groups.

### Analysis of factors associated with MOG-IgG positive in ADEM children

To further study the related factors for seropositive MOG-IgG ADEM patients, all factors with *P* < 0.05 in univariate analyses and other potentially related factors were included in the binary logistic regression analysis. Significant differences in the disease duration, meningeal involvement, and frontal lobe involvement were found between the two groups (*P* < 0.05), indicating that MOG-IgG positive ADEM patients were predicted by the longer disease duration, more meningeal involvement, and higher frontal lobe involvement (Table 3).

**Table 3:** Associations of clinical indicators with MOG-IgG-positive ADEM patients.

Factors	MOG-IgG-positive group *n* = 35	MOG-IgG-negative group *n* = 50	B	*p* value	OR	95% CI
Gender, male, n (%)	21 (60)	28 (56)	0.032	0.970	1.03	(0.193, 5.509)
Onset age, months, median (range)	69 (20–138)	76 (13–178)	−0.007	0.575	0.993	(0.970, 1.017)
Disease duration, days, median (range)	10 (1–30)*	6 (0.5–30)	0.168	0.008	1.183	(1.045, 1.339)
Meningeal involvement, n (%)	11 (31.4)*	4 (8)	4.445	0.001	85.158	(6.199, 1169.853)
Frontal lobe involvement, n (%)	29 (82.9)*	29 (58)	2.703	0.027	51.824	(2.867, 936.883)
Relapse, n (%)	5 (14.3)	1 (2)	2.978	0.106	19.639	(0.533, 723.340)
Serum TNF, (pg/ml), median (range)	1.7 (1–12.4)	2.2 (1–34)	−0.147	0.160	0.863	(0.703, 1.060)
Serum IFN-γ, (pg/ml), median (range)	1.3 (1–9.4)	3 (1–64)	−0.114	0.581	0.893	(0.596, 1.336)

*ADEM* acute disseminated encephalomyelitis, *MOG* myelin oligodendrocyte glycoprotein, *MOG-IgG-positive* ADEM patients with positive anti-MOG antibodies in serum, *MOG-IgG-negative* ADEM patients without positive anti-MOG antibodies in serum, *TNF* tumor necrosis factor, *IFN-γ* interferon γ *OR* odds ratio, *95% CI* 95% confidence interval. Binary logistic regression was used for multivariate analyses.

*indicates MOG-IgG-positive group vs. MOG-IgG-negative group, *p* < 0.05.

## Discussion

ADEM is a common phenotype of MOGADs in children. The presence of MOG antibodies in ADEM was higher than that in other demyelinating disorders [7]. Previous data showed that serum MOG-IgG was identified in 30–65% of children with ADEM [7–9]. We confirmed that 41.2% (35/85) of ADEM children were seropositive for MOG-IgG in our cohort. In the present study, MOG-IgG-positive ADEM children had a median age of 69 months, with a high incidence in winter or spring, which was in line with the previous literatures [7–10]. Although the clinical manifestations of ADEM children with positive serum MOG-IgG were similar to those without, there were still some clues that helped us to differentiate ADEM patients with positive serum MOG-IgG. There was a higher likelihood of MOG antibody positivity in younger patients or patients suffering seizures from previous reports [11, 12]. Nevertheless, we did not find the similar results. On the other hand, we found that the ADEM patients with positive serum MOG-IgG had a longer disease duration than those without, suggesting that the disease course of MOG-IgG-positive ADEM patients was more protracted. Interestingly, meningeal involvement was more common in ADEM children with MOG antibodies (31.4%, 11/35), which was higher than the 12% reported in a previous study [13]. Furthermore, a longer disease duration and meningeal involvement were independent variables associated with MOG-IgG-positive ADEM. Therefore, we demonstrated that ADEM children with positive serum MOG-IgG were more likely to have meningeal involvement and a longer disease duration, and much more attention should be given to the detection of MOG-IgG in pediatric patients with ADEM and prolonged disease duration or meningeal involvement.

CSF protein is usually normal or mildly elevated in MOG-positive ADEM patients [13]. Increased CSF protein levels were observed in 23–66% of cases [14]. In another report, all MOG-positive ADEM children with neurological sequelae (5/20) had elevated protein levels, while only 13% had no neurologic sequelae (2/15) [15]. In our cohort, the CSF protein concentrations ranged from 388 to 972 mg/L and protein elevation was observed in 13 (37.1%) cases, which was consistent with the previous data [14, 15]. Some authors argued that the cut-off of the CSF protein should be elevated, as the normal values of CSF proteins in children are lower than those in adults [14]. We believe that the normal CSF protein concentration should be related to the patient’s age, higher in the neonatal period, and then gradually decreases in the first year of life. The accurate upper normal limit of protein concentration varies depending on the technology and testing laboratory [16]. In our study, the age of onset ranged from 20 to 138 months, indicating that our patients were all over one year old. Therefore, we still used the cutoff value of 400 mg/l in the data.

MRI is an important means to diagnose ADEM. In the current study, the manifestations of cranial MRI of MOG-IgG-positive ADEM patients were consistent with the previous reports [5, 17], including multifocal irregular and asymmetric clusters and flaky lesions mainly in the subcortical white matter, accompanied by bilateral involvement of the brainstem, thalamus, and basal ganglia. It was reported that large and bilateral lesions and longitudinally extensive transverse myelitis (LETM) lesions were more common and more likely to resolve in MOG antibody-associated ADEM [18, 19]. In contrast, there was no significant difference in bilateral lesions, spinal cord involvement, or infratentorial lesions between the MOG-IgG-positive ADEM and MOG-IgG-negative ADEM children in our cohort. MRI follow-up also revealed that ADEM children with or without MOG antibodies did not differ in the resolution of the initial lesions. In our cohort, frontal lobe involvement was more likely to occur and was an independent associated factor for MOG-IgG-positive ADEM, as described previously, which may be related to the abundant blood supply of the frontal lobe [5, 17]. Thus, we concluded that it was valuable to consider frontal lobe involvement as a diagnostic clue for children who presented with ADEM due to MOG-IgG seropositivity. In our cohort, frontal lobe involvement did not show appropriate clinical alterations more often because the behavior problems were not differed between the two groups (*P* = 0.152). Other symptoms related to frontal lobe involvement were not investigated in our cohort and need a more intensive study.

The exact pathogeneses of ADEM and MOG-IgG-mediated ADEM have not been fully elucidated. Cytokines and chemokines play important roles in the progression of ADEM. A previous study showed that the acute phase of ADEM is mainly related to T helper cell (Th) 1-related cytokine abnormalities, such as significant increases in TNF, IFN-γ, IL1, IL6, and IL8 levels, and the remission period of ADEM is mainly related to Th2-related increases in IL4, IL10, and transforming growth factor-beta [20]. In this study, the serum TNF and IFN-γ levels in the MOG-IgG positive ADEM group in the acute phase were significantly lower than those in the MOG-IgG negative ADEM group, while no significant difference in the CSF cytokines was found between the two groups, suggesting that the changes in serum Th1-related cytokines were more pronounced in ADEM children without antibodies. Although some in vitro experiments have confirmed that MOG-IgG mediates cell death through the complement pathway [1, 21], no significant differences in complements were found between the two groups in the present study. Further in vitro and in vivo research on the involvement of cytokines and complements in ADEM, especially the pathogenesis of anti-MOG antibody-positive ADEM in pediatric patients, is needed.

The live cell-based MOG-IgG assay is a useful diagnostic tool for the determination of MOGAD, but sometimes it will lead to the potential for false positive results [22]. Previous studies showed that the positive predictive value (PPV) for MOG-IgG increased depending on the growth of the serum MOG-IgG titre cut-off. A study at the Mayo Clinic found a PPV of 72% using a live cell-based assay with a titre cut-off of 1:20 [23]. Another report demonstrated that live MOG-IgG CBAs showed excellent agreement for high positive samples but were more frequently discordant for low positive samples at 3 international testing centers [24]. Therefore, an argument can be made that using a high titre threshold will lead to missed diagnosis while using a low titre threshold will lead to false positives. When we make the diagnosis of MOGAD, in addition to MOG-IgG seropositivity, supplementary clinical interpretation is needed. In our cohort, we used a serum MOG-IgG titre cut-off of > 1:10, that this low cut off may lead to false positives and cause potential selection bias. Interestingly, of these 35 positive patients, 51.4% (18/35) still had seropositive MOG-IgG and 94.3% (33/35) were identified as true ADEM patients, while the other two were diagnosed as NMOSD or ADEM-ON patients during the follow-up period. This disagreement between the low cut off and the correct clinical context may be due to the high expression of serum MOG-IgG antibodies in ADEM patients. Other scholars also conducted that the presence of low positive MOG IgG was only meaningful in the correct clinical context such as in patients with ON, myelitis, ADEM, or encephalitis [24].

It has also been reported that a relapsing disease course is more likely in patients with higher MOG-IgG titres at onset and persisting MOG-IgG over time, whereas transient low titres of MOG-IgG are typically associated with a monophasic disease course, not only in ADEM patients but also in other phenotypes [25]. However, this conclusion was not consistent with our results. We found that relapsing ADEM was not correlated with the onset MOG-IgG titres or the persisting MOG-IgG, which may be due to the low cut-off of the serum MOG-IgG titre in our group. Future studies with higher MOG-IgG titres are needed.

In accordance with previous reports [8, 26], the prognosis of ADEM children with MOG antibodies was good in this study, and only 14.3% of ADEM children with positive serum MOG-IgG suffered from neurological sequelae. However, a previous study demonstrated that the recurrence rate of ADEM was as high as 1/3 [10], and patients with persistent anti-MOG seropositivity were more likely to have a multiphasic course of ADEM [8, 27, 28]. Long-term follow-up showed that children with primary ADEM might relapse during follow-up with MDEM, ON, or NMOSD [19, 27]. In this study, a total of five ADEM patients with anti-MOG seropositivity had a recurrence, with a recurrence rate of 14.3%, which was significantly higher than that in patients without serum MOG antibodies, suggesting that MOG-IgG positivity may be a risk factor for recurrence in ADEM children. Among the recurrence cases, relapse was more common in females (80%, 4/5), with a recurrence interval of less than one year, which was consistent with previous reports [27, 29]. However, another report showed that a second demyelination event might take up to 4 years to develop [27]. Further observation is needed to confirm whether the recurrence rate of ADEM will continue to increase over time.

Our study has limitations. Firstly, as a retrospective study with a small sample size, it will lead to potential bias. To obtain an intensive idea of the significance of our findings, the powers were estimated by using G*Power software. Based on an effect size = 0.5, sample sizes of N1 = 35, N2 = 50 subjects, and *P* = 0.05, the estimated power in our cohort was 72% for the t-test, 78% for the chi-square test, and 66% for the regression analysis. This low power, which was thought to be correlated with the small sample size, indicated that it may lead to false negative results, especially in the regression analysis. Studies with sufficient sample sizes are needed to improve the conclusions.

Secondly, the serum MOG-IgG titre cut-off was lower than that in some previous cohorts. As some scholars mentioned, the low cut-off may vanish the PPV of the serum MOG-IgG test and lead to false positive cases. In our data, the low cut-off may also lead to false positive ADEM, which might be corrected by reevaluating of the diagnosis in the follow-up period.

In addition, other limitations may include: This was a single-center study; thus, the findings of this study may not apply to all ADEM children seropositive for MOG-IgG in China; MRI scans were done basing the clinical symptoms, the T1, T2 and Flair sequences were scheduled in every patient but the DWI or enhanced MRI sequences were not available in some patients; this study was a retrospective study and the data of children were retrospectively collected, which may lead to a potential for selection bias. Despite these limitations, we believe that our findings provide some useful information for better understanding the clinical characteristics and related factors of ADEM children with MOG antibodies.

## Conclusion

To summarize, MOG-IgG positive ADEM frequently occurred in winter and spring, and its main clinical manifestations are encephalopathy and multifocal neurologic signs, usually with good prognosis, while some suffered from mild neurological sequelae. Compared to the MOG-IgG-negative ADEM patients, MOG-IgG-positive ADEM children had a more prolonged disease duration, more meningeal involvement, more frontal lobe involvement, and less serum TNF and INF-γ, and were more prone to recurrence.

## Data Availability

The datasets used or analyzed during the current study are not publicly available due to patient confidentiality but are available from the corresponding author on reasonable request.

## Abbreviations

ADEM: Acute disseminated encephalomyelitis
AQP4: Anti-aquaporin 4
CBAs: Cell-based assays
CNS: Central nervous system
CSF: Cerebrospinal fluid
EEG: Electroencephalogram
IFN-γ: Interferon γ
IgG: Immunoglobulin G
IL: Interleukin
LETM: Longitudinally extensive transverse myelitis
MDEM: Multiphasic disseminated encephalomyelitis
MOG: Myelin oligodendrocyte glycoprotein
MOGADs: Myelin oligodendrocyte glycoprotein-immunoglobulin G-associated disorders
MRI: Magnetic resonance imaging
NMOSD: Neuromyelitis optica spectrum disorder
ON: Optic neuritis
PPV: Positive predictive value
Th: T helper cell
TNF: Tumor necrosis factor
